# Metabolomic profiling reveals novel biomarkers of alcohol intake and alcohol-induced liver injury in community-dwelling men

**DOI:** 10.1007/s12199-015-0494-y

**Published:** 2015-10-12

**Authors:** Sei Harada, Toru Takebayashi, Ayako Kurihara, Miki Akiyama, Asako Suzuki, Yoko Hatakeyama, Daisuke Sugiyama, Kazuyo Kuwabara, Ayano Takeuchi, Tomonori Okamura, Yuji Nishiwaki, Taichiro Tanaka, Akiyoshi Hirayama, Masahiro Sugimoto, Tomoyoshi Soga, Masaru Tomita

**Affiliations:** Department of Preventive Medicine and Public Health, School of Medicine, Keio University, 35 Shinanomachi, Shinjuku-ku, Tokyo, 160-8582 Japan; Institute for Advanced Biosciences, Keio University, Tsuruoka, 997-0052 Japan; Faculty of Environment and Information Studies, Keio University, Fujisawa, 252-0882 Japan; Division of Environmental and Occupational Health, Department of Social Medicine, Faculty of Medicine, Toho University, Ota-ku, Tokyo, 143-0015 Japan

**Keywords:** Metabolomics, Alcohol, Alcoholic liver disease, Biomarker, Amino acids

## Abstract

**Objective:**

Metabolomics is a promising approach to the identification of biomarkers in plasma. Here, we performed a population-based, cross-sectional study to identify potential biomarkers of alcohol intake and alcohol-induced liver injury by metabolomic profiling using capillary electrophoresis-mass spectrometry (CE-MS).

**Methods:**

Fasting plasma samples were collected from 896 Japanese men who participated in the baseline survey of the Tsuruoka Metabolomics Cohort Study, and 115 polar metabolites were identified and absolutely quantified by CE-MS. Information on daily ethanol intake was collected through a standardized, self-administered questionnaire. The associations between ethanol intake and plasma concentration of metabolites were examined. Relationships between metabolite concentrations or their ratios and serum liver enzyme levels in the highest ethanol intake group (>46.0 g/day) were then examined by linear regression analysis. Replication analysis was conducted in 193 samples collected from independent population of this cohort.

**Results:**

Nineteen metabolites were identified to have an association with daily alcohol consumption both in the original and replication population. Three of these metabolites (threonine, glutamine, and guanidinosuccinate) were found to associate well with elevated levels of serum liver enzymes in the highest ethanol intake group, but not in the non-drinker group. We also found that the glutamate/glutamine ratio had a much stronger relation to serum γ-glutamyltransferase, aspartate transaminase, and alanine transaminase than glutamate or glutamine alone (standardized beta = 0.678, 0.558, 0.498, respectively).

**Conclusions:**

We found 19 metabolites associated with alcohol intake, and three biomarker candidates (threonine, guanidinosuccinate and glutamine) of alcohol-induced liver injury. Glutamate/glutamine ratio might also be good biomarker.

**Electronic supplementary material:**

The online version of this article (doi:10.1007/s12199-015-0494-y) contains supplementary material, which is available to authorized users.

## Introduction

Alcoholic liver disease is a worldwide burden, with 493,300 deaths and 14,544,000 disability-adjusted life years (DALYs) attributed to alcoholic liver disease worldwide in 2010, accounting for 0.9 % of all deaths and 0.6 % of all DALYs in that year [1]. Liver diseases have the highest alcohol-attributable fractions of any disease besides alcohol disorders and fetal alcohol syndrome, and alcohol consumption contributes to 50 % of the disease burden of liver cirrhosis [2]. Liver enzymes such as γ-glutamyltransferase (γ-GTP) and aminotransferase were known as biomarkers of alcohol consumption and alcoholic liver injury. However, further study is needed to clarify pathological or metabolic alterations leading to liver injury induced by alcohol intake specifically. Identification of novel biomarker candidates for monitoring of alcohol consumption or detecting signs of alcoholic liver disease could aid in the design of effective prevention programs.

Global metabolomic profiling [3, 4] in human plasma is a powerful tool for the comprehensive identification of metabolic alterations caused by chronic alcohol consumption and ensuing alcoholic liver diseases. The metabolomic profile reflects cellular activities affected by any and all possible genetic or environmental factors and facilitates identification of potential biomarkers of alcohol intake and alcohol-induced liver diseases [5, 6]. To our knowledge, the only study of alcohol-induced metabolomic differences in humans to date focused primarily on lipid metabolism [7]. However, development of alcohol-induced liver injury is also accompanied by alterations in carbohydrate and amino acid metabolism, reflecting the metabolic consequences of alcohol-induced oxidative stress [8–10]. Among several methods of metabolomic profiling, the capillary electrophoresis-mass spectrometry (CE-MS) method has high separation efficiency and compound identification capability, which allows the absolute quantification of global polar metabolites, including carbohydrates and amino acids [11–14], while being unsuitable for non-polar metabolites including most of lipid metabolites. We thus expected to add new insights into alcohol-induced metabolomic alterations with polar metabolites.

We aimed to find metabolomic biomarker candidates of alcohol intake and alcohol-induced liver injury based on the effect on plasma profiles of global polar metabolites. Here, we performed a cross-sectional study with CE-MS-based metabolomic profiling in a large, community-based population.

## Materials and methods

### Study population

The study base was 1017 men and 1109 women aged 35–74 years participating in the ongoing Tsuruoka Metabolomic Cohort Study, initiated in April 2012 in Tsuruoka City (Yamagata Prefecture, Japan). The baseline period is 3 years, during which we aim to enroll 10,000 subjects aged 35–74 years from among participants in annual municipal or worksite health check-up programs in the city. The total population of Tsuruoka City at initiation was 136,623, of whom 71,868 were aged 35–74 years. Plasma metabolomic profiling in the 1017 men and 1109 women who consented to participate (participation rate 90 % among health check-up program attendees) in the first 3 months (from April to June 2012) was completed by the end of 2013. Proportion of regular drinkers was lower in women than in men (23.8 vs. 74.2 %), as was daily ethanol intake (median 8.0 vs. 35.7 g/day). Due to the small number of female participants who were regular drinkers, only male participants were ultimately included in analysis.

To confirm associations observed in the original dataset, replication analysis was performed using independent 215 plasma samples. These replication samples were collected from subsequent male participants of our cohort study between July and August 2012.

The study was approved by the Medical Ethics Committee of the School of Medicine, Keio University, Tokyo, Japan (Approval No 20110264). Informed consent was obtained from all individual participants included in the study in written form.

### Data and sample collection

All data and samples were obtained during the annual health check-up, including blood and urine specimens. Information on drinking, smoking, diet, stress, and physical activity was collected through a standardized self-administered questionnaire.

Alcohol intake per week was calculated from the frequency of alcohol consumption during a typical week and the total alcohol intake on each occasion, and then divided by seven to obtain average alcohol intake per day [15]. Subjects were then classified as never drinkers, ex-drinkers, or current drinkers. Current drinkers were defined as subjects consuming one or more grams of ethanol per day on average.

To avoid variation due to fasting state and circadian rhythm, blood samples were collected in the morning between 8:30 am and 10:30 am after overnight fasting. Plasma samples were collected with ethylenediaminetetraacetic acid-2Na as an anticoagulant and kept at 4 °C immediately after collection. The plasma samples were centrifuged for 15 min (1500 g at 4 °C) within 1 h of collection, divided into aliquots, and kept for a maximum of 6 h at 4 °C until extraction of metabolites. Serum samples were collected with serum-separating medium and kept at room temperature after collection. Levels of γ-GTP, serum aspartate transaminase (AST), and alanine transaminase (ALT) were measured via the Japan Society of Clinical Chemistry transferable national standardized method, with γ-GTP levels measured via a colorimetric method and AST and ALT via an ultraviolet spectrophotometric method.

### Metabolomics measurement

Non-targeted mass spectrometry-based metabolomic profiling was performed with fasting plasma samples via capillary electrophoresis time-of-flight mass spectrometry (CE-TOFMS). Metabolite extraction from plasma was completed within 6 h after collection to minimize the effect of metabolic change in plasma. The extraction method has been described in detail elsewhere [16].

CE-TOFMS analysis of cationic metabolites and anionic metabolites was performed as described previously [12, 13]. The raw data were processed using our proprietary software (MasterHands) [12, 17]. As a preliminary study, we identified 290 metabolite peaks (131 cations and 159 anions) in plasma; 154 known with standard compounds and 136 unknown. We decided to routinely measure absolute concentrations of 115 metabolites (63 cations and 52 anions) a priori that were expected to be stably observed in most human plasma samples and had matched standards.

### Statistical analysis

To eliminate the possibility of liver damage caused by diseases other than alcohol liver diseases, we excluded 99 subjects who had any self-reported history of cancer, positive results on examination for hepatitis B virus surface antigen, or hepatitis C virus antibody from the original population. We also excluded 16 subjects without correctly evaluated alcohol consumption, two whose metabolome were outliers in the primary component analysis, and four without overnight fasting. We excluded 22 subjects from the replication population for the same reasons. The final dataset included 896 men in the original population and 193 men in the replication population with complete fasting plasma metabolomics measurement and estimated daily alcohol intake data.

For metabolomics data, six of 115 metabolites detected in less than 1 % of subjects were excluded from further analysis. The remaining 107 metabolite concentrations were treated as continuous variables, and 49 metabolites of them were log-transformed according to the shape of distribution.

We classified the subjects into four groups by tertile according to current amount of daily alcohol intake: non-drinkers and low, middle, and high alcohol intake groups (low group: 1.0–24.9 g/day, middle group: 25.0–46.0 g/day, high group: 46.1–205.1 g/day). To examine the association between daily alcohol intake and 107 metabolite concentrations as continuous variables (i.e., biomarkers of alcohol intake), we performed linear regression analysis between alcohol intake groups (1: non-drinkers, 2: low, 3: middle 4, high alcohol intake) and each metabolite concentration. We calculated difference between non-drinkers and high alcohol intake group for metabolites treated as normal variables, and calculated fold change from non-drinkers to high alcohol intake group for metabolites treated as log-transformed variables, using beta of the linear regression analysis. To adjust for multiple comparison, we presented *p* values using Benjamini and Hochberg’s false discovery rate (BH-FDR) method (*α* = 0.05). We also examined the association between alcohol intake and high-density lipoprotein (HDL) -cholesterol, which is well-known traditional alcohol related biomarker [18–22], to compare with the metabolite associations discovered in our study. Low-density lipoprotein (LDL) -cholesterol and triglyceride were also examined for the same reason, though associations of these and alcohol intake vary by studies [19]. In order to confirm whether found associations (FDR *p* < 0.05) of alcohol intake and the metabolites in the original dataset could be replicated in a different dataset, linear regression analysis was performed in the replication population. As a sensitivity analysis, we also performed the linear regression analysis between each metabolite concentration and alcohol intake (g/day) as a continuous variable, excluding top 5 percent drinkers (>92.0 g/day alcohol intake) as outliers.

To further explore whether the identified alcohol intake biomarkers, whose associations were replicated, were associated with alcoholic liver injury or not after controlling for age, we performed linear regression analyses in the highest tertile group of daily alcohol consumption between alcohol intake-related metabolites and serum liver enzymes, including γ-GTP, AST, and ALT. To adjust for multiple comparison, we calculated *p* values adjusted using the BH-FDR method (*α* = 0.05). The same analyses were done in the non-drinker group to examine the possibility that some metabolites were related to an increase in liver enzymes due to non-alcoholic liver diseases. We also examined the association between glutamate/glutamine ratio on serum γ-GTP, AST, and ALT via linear regression analysis because this ratio was reported to correlate well with γ-GTP in patients of alcoholic liver diseases in the previous clinical study [23]. The metabolites associated with alcoholic liver injury in the original dataset (FDR *p* < 0.05) were also examined in the replication population.

Sensitivity analysis was also performed in each analysis by incorporating possible confounders into the constructed models as continuous variables: age, BMI, smoking numbers per year, systolic blood pressure, HDL-cholesterol, and hemoglobin A1c. Daily dietary energy intake, and daily physical activity were also adjusted in the full model. We used SAS 9.3 (SAS Institute Inc., Cary, NC) for all statistical analyses.

## Results

### Characteristics

Table 1 shows the characteristics of the four groups by alcohol intake in the original population. No marked differences between groups were observed in body mass index, fasting glucose, or hemoglobin A1c. Further, no obvious difference was noted in ALT levels. In contrast, γ-GTP and AST levels were elevated with increasing alcohol intake, as were systolic blood pressure, diastolic blood pressure, triglycerides, HDL-cholesterol, and the percentage of participants with high daily physical activity, while LDL-cholesterol and the percentage of participants with high dietary energy intake decreased with increasing alcohol intake. The characteristics of the replication population were similar to the original population, as shown in eTable 1.

**Table 1:** Characteristics of original population

Variable	Original population			
Alcohol intake	Non-drinker (*n* = 231)	Low (*n* = 220)	Middle (*n* = 219)	High (*n* = 226)
Alcohol intake (g/day)^a^	N.A.	12.0 (1.0–24.6)	35.7 (25.0–46.0)	67.2 (46.1–205.1)
Age (years)^b^	62.5 (8.4)	63.0 (8.4)	62.6 (7.2)	62.0 (7.1)
Body mass index (kg/m^2)b^	23.8 (3.3)	23.4 (3.0)	23.5 (2.8)	23.4 (3.0)
Hypertension^1^, Yes	44.2 % (102/231)	47.7 % (105/220)	53.9 % (118/219)	59.3 % (134/226)
On medication, Yes	34.6 % (80/231)	31.4 % (69/220)	33.8 % (74/219)	38.9 % (88/226)
SBP (mmHg)^c^	123.9 (84–186)	129.9 (96-185)	132.4 (95–189)	133.8 (96–212)
DBP (mmHg)^c^	74.3 (51–106)	77.7 (47–113)	79.5 (54–112)	80.4 (56–109)
IGT^2^, Yes	24.2 % (56/231)	28.9 % (63/218)	26.0 % (57/219)	28.3 % (64/226)
On medication, Yes	10.4 % (24/231)	10.5 % (23/220)	9.6 % (21/219)	9.7 % (22/226)
FPG (mg/dL)^c^	100.8 (76–213)	103.8 (81–200)	102.6 (80–205)	103.9 (64–211)
HbA1c (%)^c^	5.8 (4.9–8.7)	5.8 (5.0–8.9)	5.7 (4.8–9.2)	5.7 (5.0–9.4)
Dyslipidemia^3^, Yes	55.8 % (129/231)	48.6 % (107/220)	40.2 % (88/219)	46.5 % (105/226)
On medication, Yes	19.9 % (46/231)	14.1 % (31/220)	12.8 % (28/219)	11.5 % (26/226)
Total cholesterol (mg/dL)^b^	201.1 (34.4)	204.5 (33.1)	205.4 (32.5)	205.4 (33.4)
LDL cholesterol (mg/dL)^b^	121.4 (30.2)	119.8 (29.1)	114.4 (29.4)	111.9 (30.5)
HDL cholesterol (mg/dL)^b^	57.4 (13.3)	62.3 (13.9)	68.1 (16.5)	69.0 (18.3)
Triglyceride (mg/dL)^c^	98.9 (25–447)	100.4 (41–872)	101.1 (30–564)	103.8 (37–1879)
AST (lU/L)^c^	22.3 (12–85)	23.6 (12–282)	24.7 (14–100)	27.2 (14–132)
ALT (lU/L)^c^	20.0 (7–92)	21.4 (7–145)	21.1 (5–91)	22.4 (8–98)
γ-GTP (lU/L)^c^	24.7 (10–477)	33.7 (10–913)	38.9 (12–428)	54.7 (13–1295)
Smoking, Yes	26.8 % (62/231)	20.0 % (44/220)	29.7 % (65/219)	36.7 % (83/226)
Ex	46.8 % (108/231)	52.3 % (115/220)	56.2 % (123/219)	52.7 % (119/226)
High daily activity^d^, Yes	20.0 % (45/225)	19.6 % (43/219)	28.9 % (63/218)	31.2 % (69/221)
High dietary intake^d^, Yes	29.0 % (67/231)	25.0 % (55/220)	26.0 % (57/219)	19.9 % (45/226)

*ALT* alanine aminotransferase, *AST* aspartate aminotransferase, *γ-GTP* gamma-glutamyl transpeptidase, *DBP* diastolic blood pressure, *FPG* fasting plasma glucose; *HDL* high-density lipoprotein, *IGT* impaired glucose tolerance, *LDL* low-density lipoprotein, *SBP* systolic blood pressure

^a^Reported as median (range)

^b^Reported as mean (standard deviation)

^c^Reported as geometric mean (range)

^d^Percent and numbers of the highest quantile are shown

^1^Hypertension: Systolic blood pressure ≥ 140 mmHg, diastolic blood pressure ≥90 mmHg or on medication

^2^Impaired glucose tolerance: Glucose ≥ 110 mg/dL, hemoglobinA1c ≥ 6.5 % or on medication

^3^Dyslipidemia: Triglyceride ≥ 150 mg/dL, LDL cholesterol ≥ 140 mg/dL, HDL cholesterol ≤ 40 mg/dL or on medication

### Association between alcohol intake and metabolome

**Fig. 1:**
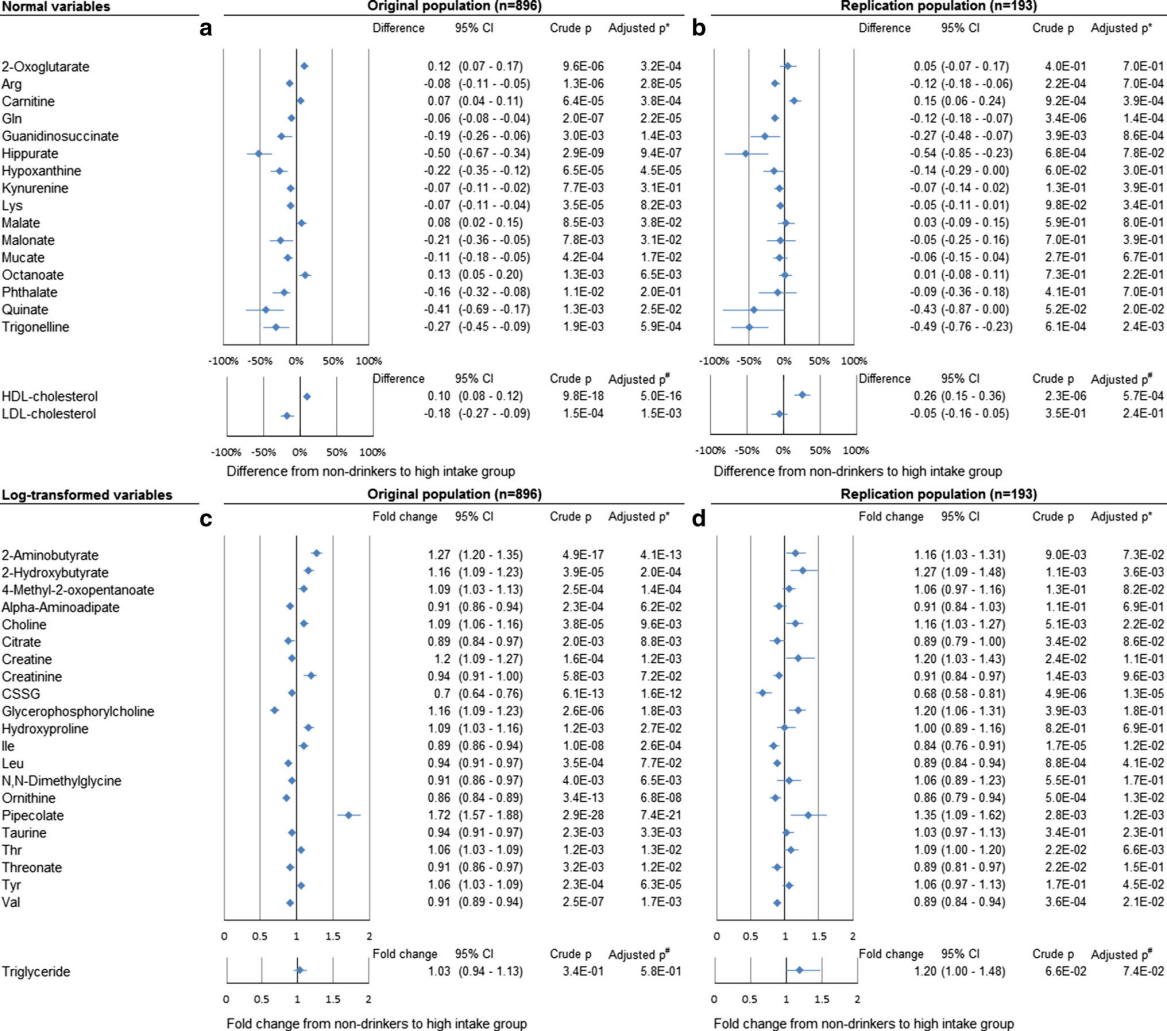
The associations between plasma metabolites and alcohol intake groups. The associations between plasma metabolites and alcohol intake groups (1: non-drinkers, 2: low 3: middle 4: high alcohol intake groups) in the original (**a**, **c**) and the replication population (**b**, **d**). Linear regression analysis between each metabolite and alcohol intake group was performed (*p* values are shown); then difference between non-drinkers and the high alcohol intake group for normal variables (**a**, **b**) and fold change for log-transformed variables (**c**, **d**) were calculated using beta of the linear regression analysis. The metabolites with less than 0.05 FDR *p* values in the original population were shown in this figure (**a**, **c**). Replication analyses were performed for only these metabolites (**b**, **d**). *CI* Confidence interval, *CSSG* Cysteine-glutathione disulfide, *FDR* False discovery rate, *HDL* High-density lipoprotein, *LDL* Low-density lipoprotein. *Adjusted for age, BMI, smoking numbers per year, systolic blood pressure, HDL-cholesterol, hemoglobin A1c, daily dietary energy intake, and daily physical activity. ^#^Adjusted for age, BMI, smoking numbers per year, systolic blood pressure, hemoglobin A1c, daily dietary energy intake, and daily physical activity

In total, 36 polar metabolites related to alcohol consumption even after adjusted for age. (FDR *p* < 0.05) (Fig. 1; results of all metabolites in eTables 2, 3). Twenty-seven metabolites still showed *p* values less than 0.05 in full-adjusted model. Exclusion of ex-drinkers (*n* = 62) from the non-drinker group also did not change the results substantially.

Of the 27 metabolites associated with alcohol consumption after full adjustment, 19 associations (70.4 %) were confirmed in the replication set (*p* < 0.05). Thirteen metabolites were involved in amino acid metabolism, three in carbohydrate metabolism, two in lipid metabolism, and one in cofactor and vitamin metabolism. Various amino acid pathways were associated with alcohol consumption, including branched chain amino acids, arginine, threonine, and glutamine. These results were similar in the sensitivity analysis to examine the association between each metabolite concentration and alcohol intake (g/day) as a continuous variable, instead of the alcohol intake group score.

As common lipid biomarkers, the results of HDL-cholesterol, LDL-cholesterol, and triglyceride were also analyzed. HDL-cholesterol was associated with alcohol intake as well known. LDL-cholesterol also was associated, but this association was not replicated. Triglyceride had nearly one fold change. The magnitude of difference seemed to be stronger than HDL-C or LDL-C in some of metabolites such as hippurate and pipecolate, while being similar in most metabolites.

### Association between alcohol-related metabolites and serum γ-GTP, AST, and ALT elevation

**Fig. 2:**
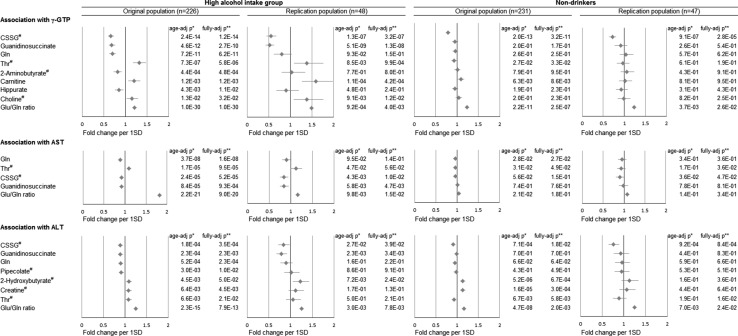
The associations between alcohol-related plasma metabolites and serum γ-GTP, AST and ALT. The associations between alcohol-related plasma metabolites and serum γ-GTP, AST and ALT in the high alcohol intake group and non-drinkers were shown. Linear regression analysis between each alcohol-related metabolite (log-transformed if necessary) and γ-GTP, AST, and ALT (log-transformed) was performed (*p* values are shown); then we calculated fold change and 95 % confidence interval of serum γ-GTP, AST, and ALT per one standard deviation increase in each metabolite using standardized beta of the linear regression analysis. The metabolites with less than 0.05 false discovery rate p-values in the original population and glutamine/glutamine ratio were shown in this figure. Replication analyses were performed for only these variables. *ALT* alanine aminotransferase, *AST* aspartate aminotransferase, *CSSG* Cysteine-glutathione disulfide, *γ-GTP* gamma-glutamyl transpeptidase, *SD* standard deviation. ^#^log-transformed variables. *Adjusted for age. **Adjusted for age, BMI, smoking numbers per year, systolic blood pressure, HDL-cholesterol, hemoglobin A1c, daily dietary energy intake, and daily physical activity

Figure 2 and eTables 4–6 indicate the results of the linear regression analyses of the association between alcohol-related metabolites and γ-GTP, AST, and ALT levels among the high alcohol intake group. Eight metabolites were associated with serum γ-GTP in the original set, and six metabolites’ [threonine, guanidinosuccinate, glutamine, choline, carnitine, and cysteine-glutathione disulfide (CSSG)] associations were also observed in the replication set. These results remained mostly unchanged even after adjustment for possible confounders. Threonine, guanidinosuccinate, glutamine, and CSSG also related to serum AST in both the original set and the replication set. Because elevation of serum γ-GTP and AST in drinkers often reflects alcoholic liver injury [24] threonine, guanidinosuccinate, glutamine, choline, carnitine, and CSSG metabolites could be considered to have an association with alcoholic liver injury.

To determine whether similar associations were observable or not under ‘non-drinking’ conditions, we also performed linear regression analyses in the non-drinker group (Fig. 2; eTables 4–6). Threonine and 2-aminobutyrate (2AB) levels clearly differed from those in the high-intake group. Threonine had negative associations with serum γ-GTP and AST levels among non-drinkers but positive associations in the high-intake group. These observations were also found in the replication set. Further, there was no association between plasma 2AB level and γ-GTP level in the non-drinker group while a negative association was observed in the high-intake group, although it was not replicated. Guanidinosuccinate, glutamine, and choline which showed associations (FDR *p* < 0.05) in the high-intake group also had no association in the non-drinkers. In contrast to these metabolites, CSSG had strong associations with serum liver enzymes in both the non-drinker and high-intake groups, indicating that the observed associations were likely due to factors other than alcohol intake. These results were consistent even after excluding ex-drinkers from non-drinkers.

## Discussion

In the present study, we revealed the metabolomic differences induced by alcohol intake and found new biomarker candidates of alcohol-induced liver injury in human plasma using CE-MS-based global metabolomic profiling among community-dwelling men. Plasma concentrations of 27 metabolites were associated with alcohol consumption after being adjusted for possible confounders (FDR *p* < 0.05), and 19 metabolites associations were reconfirmed in the replication set. Among the 19 metabolites, three of which (threonine, guanidinosuccinate and glutamine) were simultaneously associated with liver injury manifested by elevated serum liver enzymes among regular drinkers. Metabolomic profiling studies for alcohol-related health effects in humans are scarce. One study of alcohol-induced metabolomic difference has been previously reported, focused mainly on lipid metabolome and suggested that metabolomic profiles based on phosphatidylcholines, lysophosphatidylcholines, ether lipids, and sphingolipids form a new class of biomarkers for alcohol consumption [7]. Our results presented new insights to understand alcohol-induced alterations in broad metabolome including both of polar and non-polar metabolites, adding the findings in polar metabolites such as amino acids and carbohydrates by using CE-MS platform to known findings in lipids. To our knowledge, this is the first epidemiological study to use metabolomics to investigate potential biomarkers associated with alcohol-related liver injury.

Among the 107 polar metabolites we examined, 19 were associated with daily alcohol consumption in both the original and the replication set. In particular, changes in polar metabolite concentrations in plasma related to methionine metabolism and glutathione pathway, such as CSSG, 2AB, and choline, may reflect reactions to oxidative stress induced by high daily alcohol consumption. Plasma levels of CSSG, a biomarker of oxidative stress that is strongly related to hepatic glutathione level [25], decreased with increasing alcohol intake. Given that hepatic glutathione depletion after chronic alcohol consumption has been demonstrated in both experimental animals as well as humans [8] and that most glutathione is rapidly converted to CSSG in human plasma because of poor stability [26], low plasma CSSG levels suggest that chronic alcohol consumption may induce an oxidizing state in the liver and cause subsequent depletion of glutathione in the organ.

Plasma levels of 2AB were also strongly related to alcohol intake in our results. Acceleration of glutathione turnover due to alcohol administration might play an important role in the increase in plasma 2AB because acceleration of glutathione enhances the conversion of homocysteine to cysteine but inhibits that of homocysteine to methionine [8, 10], which results in increased production of 2AB. Our observation of higher choline and lower N,N-dimethylglycine concentrations in the drinkers’ plasma than in nondrinkers’ plasma may support the assumption that conversion of homocysteine to methionine was inhibited, as has been previously reported [10].

2AB has been extensively investigated as a biomarker of alcohol-related chronic metabolic change. In a healthy population, active drinkers without liver disease tended to have a higher serum 2AB concentration than non-drinkers [27]. In contrast, patients in a clinical study with severely developed alcohol-induced liver disease had lower serum 2AB concentration than non-drinkers [28]. Consistent with these findings, we observed that plasma 2AB level increased with increasing alcohol intake and decreased with increasing serum γ-GTP level only in the high alcohol intake group, though this finding was not confirmed in the replication set. These findings may indicate that plasma 2AB levels increase due to oxidative stress with alcohol intake in healthy people but decrease once hepatic pathological change is observed.

Of note, plasma threonine had strong positive associations with γ-GTP and AST levels only in the high alcohol intake group, while a weak negative association was observed among non-drinkers as well as low and middle alcohol intake groups. In the high alcohol intake group, geometric mean concentrations of plasma threonine were obviously different between subjects with increased AST and normal serum AST [172.9 μM for subjects with increased AST (≥40 IU/L) and 134.4 μM for those with normal serum AST (<40 IU/L); *p* < 0.0001]. On the other hand, this association was not observed in the other groups, including non-drinkers (117.4 μM for the AST ≥ 40 IU/L group and 127.9 μM for the AST < 40 IU/L group). Further, plasma threonine level exceeded 250 μM when the analysis was restricted to the high alcohol intake group with AST ≥ 100 IU/L, although this group consisted of only 2 subjects. These observations suggest that threonine may be a specific biomarker of alcohol-induced liver injury.

The mechanism by which plasma threonine is elevated among heavy drinkers with accompanying liver damage is unclear, with one possible explanation being that the main metabolic pathway of threonine in humans, catabolism to 2-oxobutyrate via serine dehydratase [29], might be inhibited, as plasma 2AB concentration, a good biomarker of alcohol drinking and one of the end products of 2-oxobutyrate, is decreased only when alcohol-induced liver injury exists. A second possibility is that intake of threonine, an essential amino acid, is increased in the high alcohol intake group; this seems unlikely, however, because essential amino acid concentrations other than threonine were lower in the high alcohol intake group than in the other groups (shown in eTables 2, 3), indicating that heavy drinkers tended to eat less. A previous study suggested that levels of both threonine and 2AB in plasma should increase when threonine intake is high [29]. Thus, threonine might be an important biomarker candidate of alcohol-induced liver injury, reflecting metabolic alterations in the liver.

Our findings that plasma glutamine concentration decreased in an alcohol intake-dependent manner, while a decrease in plasma glutamine was associated with elevation of serum liver enzymes in the high alcohol intake group only, suggest that glutamine might play an important role in protecting the liver from alcohol. These results are consistent with a previous clinical study showing that drinking patients with alcohol-induced liver diseases had low plasma glutamine levels compared to healthy people or patients with non-alcoholic liver diseases, which was alleviated after one month of alcohol abstinence [23]. This putative protective role of glutamine is supported by the finding that pretreatment with glutamine prevented ethanol-induced liver injury in mice by improving ethanol-induced inflammatory response [30]. Our observation of lower p values in glutamate/glutamine ratio suggests that alteration of glutamate and glutamine pathway is strongly related to alcoholic liver injury.

Guanidinosuccinate concentration also decreased in an alcohol intake-dependent manner. In addition, guanidinosuccinate concentration had highly negative association with elevation of serum liver enzymes only in the high alcohol intake group. This finding suggests that guanidinosuccinate declines specifically in alcohol-induced liver injury. Considering the previous report which showed that alcoholic cirrhotic patients had much lower serum guanidinosuccinate than non-alcoholic cirrhotic patients or controls [31], guanidinosuccinate could be a biomarker candidate to discriminate alcohol-induced liver injury from other liver injuries.

Although this study was performed using carefully designed epidemiological protocols to minimize measurement errors, some limitations warrant mention. First, the study was conducted under a cross-sectional design, and the temporality of cause-effect relationships is not assured. Reverse causality is likely to occur if a subject who experienced liver enzyme abnormalities changed his drinking habit. Careful follow-up and intervention studies with proper assessment of liver functions are further needed to overcome these limitations. Second, various factors might have influenced measurement variability in the metabolomic analysis, in turn potentially causing both random and biased misclassification of metabolomic data. Sampson et al. indicated that within-subject variability in assay accounted for the majority of variability in more than half of metabolites they measured using LC–MS [32]. To minimize variability in our study, we set a uniform fasting condition on study participants and standardized the quality control procedures for metabolomic analysis. Third, information was obtained as average alcohol intake for a typical day only. However, metabolic pathways might be affected by unusual alcohol intake, such as binging.

In conclusion, we found 19 metabolites different for alcohol intake, and three biomarker candidates (threonine, guanidinosuccinate and glutamine) of alcohol-induced liver injury. The glutamate/glutamine ratio might also be a good biomarker. Follow-up study of the subjects to elucidate causality is now on-going.

## Electronic supplementary material

Supplementary material 1 (DOCX 24 kb)

Supplementary material 2 (DOCX 36 kb)

Supplementary material 3 (DOCX 34 kb)

Supplementary material 4 (DOCX 31 kb)

Supplementary material 5 (DOCX 29 kb)

Supplementary material 6 (DOCX 30 kb)
