# Supplementary material for: Metabolomic profiling reveals novel biomarkers of alcohol intake and alcohol-induced liver injury in community-dwelling men
Source: Environ Health Prev Med. 2015 Oct 12;21(1):18–26. doi: 10.1007/s12199-015-0494-y (PMC4693765; doi:10.1007/s12199-015-0494-y)
Supplement: Supplementary file 1 — Supplementary material 1 (DOCX 24 kb) [file 12199_2015_494_MOESM1_ESM.docx]

| **eTable 1.** Characteristics of replication population | | | | |  |  |  |  |  |  |  |  |  |
| --- | --- | --- | --- | --- | --- | --- | --- | --- | --- | --- | --- | --- | --- |
|  |  |  |  |  |  |  |  |  |  |  |  |  |  |
| **Variable** |  |  | **Replication population** | | | | | | | | | | |
| **Alcohol Intake** |  |  | **Non-drinker (n=47)** | |  | **Low (n=46)** | |  | **Middle (n=52)** | |  | **High (n=48)** | |
| Alcohol intake^a^ | g/day |  | N.A. | N.A. |  | 10.6 | (2.0-19.9) |  | 34.3 | (20.0-46.3) |  | 62.7 | (46.4-217.1) |
| Age^b^ | years |  | 61.5 | (8.4) |  | 62.5 | (8.7) |  | 62.3 | (7.4) |  | 58.4 | (7.36) |
| Body mass index^b^ | kg/m^2^ |  | 24.7 | (2.8) |  | 24.2 | (3.4) |  | 23.8 | (2.7) |  | 24.0 | (2.9) |
| Hypertension^1^ | Yes |  | 34.0% | (16/47) |  | 47.8% | (22/46) |  | 42.3% | (22/52) |  | 62.5% | (30/48) |
| On medication | Yes |  | 23.4% | (11/47) |  | 21.7% | (10/46) |  | 28.8% | (15/52) |  | 35.4% | (17/48) |
| SBP^c^ | mmHg |  | 129.6 | (17.6) |  | 133.8 | (20.8) |  | 129.5 | (17.6) |  | 140.6 | (17.5) |
| DBP^c^ | mmHg |  | 77.8 | (10.7) |  | 81.8 | (14.2) |  | 79.3 | (9.1) |  | 83.3 | (9.0) |
| IGT^2^ | Yes |  | 25.5% | (12/47) |  | 23.9% | (11/46) |  | 32.7% | (17/52) |  | 43.8% | (21/48) |
| On medication | Yes |  | 12.8% | (6/47) |  | 10.9% | (5/46) |  | 13.5% | (7/52) |  | 12.5% | (6/48) |
| FPG^c^ | mg/dL |  | 104.7 | (70-230) |  | 105.8 | (83-162) |  | 105.6 | (83-232) |  | 108.9 | (85-149) |
| HbA1c^c^ | % |  | 6.0 | (4.9-9.3) |  | 5.7 | (5.1-7.1) |  | 5.8 | (5.1-8.7) |  | 5.7 | (4.9-7.1) |
| Dyslipidemia^3^ | Yes |  | 55.3% | (26/47) |  | 50.0% | (20/46) |  | 53.9% | (28/52) |  | 50.0% | (24/48) |
| On medication | Yes |  | 14.9% | (7/47) |  | 10.9% | (5/46) |  | 3.8% | (2/52) |  | 10.4% | (5/48) |
| Total cholesterol^b^ | mg/dL |  | 194.4 | (38.3) |  | 201.9 | (39.2) |  | 199.4 | (35.9) |  | 210.6 | (36) |
| LDL cholesterol^b^ | mg/dL |  | 114.7 | (33.2) |  | 118.3 | (32.4) |  | 110.3 | (30.6) |  | 110.8 | (34.1) |
| HDL cholesterol^b^ | mg/dL |  | 53.2 | (14.0) |  | 58.2 | (14.5) |  | 59.0 | (14.1) |  | 68.3 | (15.1) |
| Triglyceride^c^ | mg/dL |  | 121.9 | (53-285) |  | 117.6 | (52-569) |  | 129.5 | (39-392) |  | 145 | (53-565) |
| AST^c^ | lU/L |  | 22.6 | (11-131) |  | 23.5 | (14-45) |  | 24.9 | (12-47) |  | 29.2 | (15-80) |
| ALT^c^ | lU/L |  | 22.5 | (6-206) |  | 24.5 | (9-135) |  | 22.6 | (9-61) |  | 27.8 | (11-103) |
| γ-GTP^c^ | lU/L |  | 30.5 | (12-122) |  | 39.7 | (13-147) |  | 53.9 | (14-373) |  | 74.7 | (17-469) |
| Smoking | Yes |  | 31.9% | (15/47) |  | 23.9% | (11/46) |  | 44.2% | (23/52) |  | 27.1% | (13/48) |
|  | Ex |  | 53.2% | (25/47) |  | 43.5% | (20/46) |  | 38.5% | (20/52) |  | 52.1% | (25/48) |
| High daily activity^d^ | Yes |  | 11.1% | (5/45) |  | 26.1% | (12/46) |  | 28.9% | (15/52) |  | 32.6% | (15/46) |
| High dietary intake^d^ | Yes |  | 29.8% | (14/47) |  | 28.3% | (13/46) |  | 26.9% | (14/52) |  | 14.6% | (7/52) |
| ALT, alanine aminotransferase; AST, aspartate aminotransferase; γ-GTP, gamma-glutamyl transpeptidase; | | | | | | | | | | | | | |
| DBP, diastolic blood pressure; FPG, fasting plasma glucose; HDL, high-density lipoprotein; | | | | | | | | | | | | | |
| IGT, impaired glucose tolerance; LDL, low-density lipoprotein; SBP, systolic blood pressure | | | | | | | | | | | | | |
| a Reported as median (range) | | | | | | | | | | | | | |
| b Reported as mean (standard deviation) | | | | | | | | | | | | | |
| c Reported as geometric mean (range) | | | | | | | | | | | | | |
| d Percent and numbers of the highest quantile are shown. | | | | | | | | | | | | | |
| 1 Hypertension: Systolic blood pressure ≥ 140 mmHg, diastolic blood pressure ≥ 90 mmHg or on medication | | | | | | | | | | | | | |
| 2 Impaired glucose tolerance: Glucose ≥ 110 mg/dL, hemoglobinA1c ≥ 6.5% or on medication | | | | | | | | | | | | | |
| 3 Dyslipidemia: Triglyceride ≥ 150 mg/dL, LDL cholesterol ≥ 140 mg/dL, HDL cholesterol ≤ 40 mg/dL or on medication | | | | | | | | | | | | | |
