# Supplementary material for: Metabolomic profiling reveals novel biomarkers of alcohol intake and alcohol-induced liver injury in community-dwelling men
Source: Environ Health Prev Med. 2015 Oct 12;21(1):18–26. doi: 10.1007/s12199-015-0494-y (PMC4693765; doi:10.1007/s12199-015-0494-y)
Supplement: Supplementary file 2 — Supplementary material 2 (DOCX 36 kb) [file 12199_2015_494_MOESM2_ESM.docx]

| **eTable 2.** The associations between metabolites (normal variables) and alcohol intake in the original population. | | | | | | | | | |  |  |  | |  |  |  |
| --- | --- | --- | --- | --- | --- | --- | --- | --- | --- | --- | --- | --- | --- | --- | --- | --- |
|  |  |  | | | | | | | | | | | | | | |
| **Normal variables** |  |  |  |  | Crude | | |  | Age-adjusted | | |  | Fully-adjusted | | | |
|  |  | Difference | 95% CI |  | Beta | p | FDR p |  | Beta* | p* | FDR p* |  | Beta** | | p** | FDR p** |
| 1-Methylnicotinamide |  | 0.06 | (-0.06 - 0.17) |  | 0.01 | 2.6E-01 | 4.3E-01 |  | 0.01 | 2.7E-01 | 4.4E-01 |  | 0 | | 7.1E-01 | 8.0E-01 |
| 2-Oxobutyrate |  | 0.06 | (-0.15 - 0.27) |  | 0.18 | 5.6E-01 | 6.9E-01 |  | 0.21 | 5.0E-01 | 6.4E-01 |  | 0 | | 1.0E+00 | 1.0E+00 |
| 2-Oxoglutarate |  | 0.12 | (0.07 - 0.17) |  | 0.57 | 9.6E-06 | <.0001 |  | 0.58 | 6.9E-06 | <.0001 |  | 0.5 | | 3.2E-04 | 2.5E-03 |
| 2-Oxoisopentanoate |  | 0.03 | (-0.01 - 0.08) |  | 0.16 | 1.6E-01 | 3.0E-01 |  | 0.16 | 1.5E-01 | 2.8E-01 |  | 0.18 | | 1.4E-01 | 3.1E-01 |
| 3-Indoxyl sulfate |  | -0.12 | (-0.23 - -0.01) |  | -0.19 | 4.2E-02 | 1.0E-01 |  | -0.18 | 5.4E-02 | 1.3E-01 |  | -0.16 | | 1.2E-01 | 2.8E-01 |
| 4-Acetylbutyrate |  | -0.10 | (-0.20 - 0.00) |  | -0.06 | 3.5E-02 | 9.3E-02 |  | -0.07 | 3.2E-02 | 8.4E-02 |  | -0.09 | | 8.3E-03 | 3.5E-02 |
| 4-Oxopentanoate |  | -0.10 | (-0.24 - 0.04) |  | -0.14 | 1.5E-01 | 2.9E-01 |  | -0.14 | 1.5E-01 | 2.7E-01 |  | -0.17 | | 1.1E-01 | 2.7E-01 |
| 4-Pyridoxate |  | 0.00 | (-0.56 - 0.56) |  | 0 | 7.3E-01 | 7.8E-01 |  | 0 | 7.3E-01 | 8.0E-01 |  | 0 | | 5.6E-01 | 7.3E-01 |
| Asymmetric dimethylarginine |  | -0.05 | (-0.10 - 0.00) |  | -0.01 | 6.6E-02 | 1.5E-01 |  | -0.01 | 7.1E-02 | 1.6E-01 |  | -0.01 | | 5.8E-02 | 1.7E-01 |
| Adenosine diphosphate |  | -0.12 | (-0.48 - 0.24) |  | -0.01 | 6.8E-01 | 7.6E-01 |  | -0.01 | 6.8E-01 | 7.7E-01 |  | -0.02 | | 2.7E-01 | 4.7E-01 |
| Arg |  | -0.08 | (-0.11 - -0.05) |  | -2.32 | 1.3E-06 | <.0001 |  | -2.31 | 1.5E-06 | <.0001 |  | -2.19 | | 2.8E-05 | 4.0E-04 |
| Azelate |  | 0.00 | (-0.09 - 0.09) |  | 0 | 7.1E-01 | 7.7E-01 |  | 0 | 7.4E-01 | 8.0E-01 |  | 0 | | 6.3E-01 | 7.7E-01 |
| Carnitine |  | 0.07 | (0.04 - 0.11) |  | 1.56 | 6.4E-05 | 4.0E-04 |  | 1.57 | 6.1E-05 | 4.0E-04 |  | 1.53 | | 3.8E-04 | 2.7E-03 |
| cis-Aconitate |  | 0.02 | (-0.04 - 0.07) |  | 0.03 | 5.0E-01 | 6.4E-01 |  | 0.04 | 4.1E-01 | 5.6E-01 |  | 0.07 | | 1.4E-01 | 3.1E-01 |
| Citraconate |  | 0.00 | (-0.26 - 0.13) |  | 0 | 3.8E-01 | 5.3E-01 |  | -0.01 | 3.6E-01 | 5.1E-01 |  | 0 | | 6.2E-01 | 7.7E-01 |
| Cysteine S-sulfate |  | 0.04 | (-0.13 - 0.22) |  | 0.01 | 6.3E-01 | 7.5E-01 |  | 0.01 | 5.9E-01 | 7.0E-01 |  | 0.03 | | 2.5E-01 | 4.7E-01 |
| Decanoate |  | -0.01 | (-0.13 - 0.11) |  | -0.02 | 8.6E-01 | 8.9E-01 |  | -0.02 | 8.7E-01 | 9.0E-01 |  | -0.06 | | 6.2E-01 | 7.7E-01 |
| Dodecanedioate |  | 0.00 | (-0.35 - 0.35) |  | 0 | 9.7E-01 | 9.8E-01 |  | 0 | 9.3E-01 | 9.3E-01 |  | 0 | | 7.9E-01 | 8.3E-01 |
| Ethanolamine phosphate |  | -0.09 | (-0.37 - 0.23) |  | -0.02 | 5.5E-01 | 6.9E-01 |  | -0.02 | 5.7E-01 | 7.0E-01 |  | -0.02 | | 6.2E-01 | 7.7E-01 |
| Fumarate |  | 0.13 | (0.02 - 0.25) |  | 0.07 | 3.4E-02 | 9.2E-02 |  | 0.08 | 2.8E-02 | 7.8E-02 |  | 0.07 | | 7.2E-02 | 1.9E-01 |
| Gamma-Butyrobetaine |  | -0.02 | (-0.07 - 0.02) |  | -0.01 | 3.1E-01 | 4.8E-01 |  | -0.01 | 3.4E-01 | 4.9E-01 |  | -0.01 | | 3.1E-01 | 5.1E-01 |
| Gln |  | -0.06 | (-0.08 - -0.04) |  | -14.56 | 2.0E-07 | <.0001 |  | -14.31 | 2.8E-07 | <.0001 |  | -12.95 | | 2.2E-05 | 3.0E-04 |
| Glucuronate |  | -0.02 | (-0.13 - 0.10) |  | -0.02 | 7.7E-01 | 8.1E-01 |  | -0.01 | 8.5E-01 | 8.8E-01 |  | 0.02 | | 8.0E-01 | 8.3E-01 |
| Glutarate |  | 0.04 | (-0.04 - 0.13) |  | 0.07 | 3.1E-01 | 4.8E-01 |  | 0.07 | 2.8E-01 | 4.4E-01 |  | 0.08 | | 2.9E-01 | 4.9E-01 |
| Glycerophosphate |  | 0.02 | (-0.09 - 0.12) |  | 0.02 | 7.6E-01 | 8.0E-01 |  | 0.02 | 7.5E-01 | 8.0E-01 |  | 0.05 | | 3.2E-01 | 5.2E-01 |
| Gly-Gly |  | -0.32 | (-0.95 - 0.00) |  | -0.01 | 1.4E-01 | 2.8E-01 |  | -0.01 | 1.2E-01 | 2.5E-01 |  | -0.01 | | 1.5E-01 | 3.3E-01 |
| Guanidinoacetate |  | 0.02 | (-0.03 - 0.07) |  | 0.02 | 4.5E-01 | 6.0E-01 |  | 0.02 | 4.4E-01 | 5.8E-01 |  | 0.01 | | 6.9E-01 | 7.9E-01 |
| Guanidinosuccinate |  | -0.19 | (-0.26 - -0.06) |  | -0.03 | 3.0E-03 | 1.1E-02 |  | -0.02 | 4.1E-03 | 1.4E-02 |  | -0.03 | | 1.4E-03 | 8.7E-03 |
| Heptanoate |  | -0.09 | (-0.22 - 0.06) |  | -0.03 | 2.2E-01 | 3.8E-01 |  | -0.03 | 2.2E-01 | 3.9E-01 |  | -0.03 | | 2.1E-01 | 4.0E-01 |
| Hexanoate |  | 0.02 | (-0.04 - 0.09) |  | 0.04 | 4.3E-01 | 5.8E-01 |  | 0.04 | 4.3E-01 | 5.8E-01 |  | 0.04 | | 4.6E-01 | 6.6E-01 |
| Hippurate |  | -0.50 | (-0.67 - -0.34) |  | -0.85 | 2.9E-09 | <.0001 |  | -0.84 | 4.0E-09 | <.0001 |  | -0.77 | | 9.4E-07 | <.0001 |
| Homovanillate |  | -0.07 | (-0.22 - 0.09) |  | -0.06 | 4.1E-01 | 5.7E-01 |  | -0.05 | 4.5E-01 | 5.8E-01 |  | -0.05 | | 5.2E-01 | 7.1E-01 |
| Hypotaurine |  | -0.04 | (-0.30 - 0.17) |  | -0.01 | 6.4E-01 | 7.6E-01 |  | -0.01 | 6.2E-01 | 7.3E-01 |  | -0.01 | | 7.5E-01 | 8.2E-01 |
| Hypoxanthine |  | -0.22 | (-0.35 - -0.12) |  | -0.11 | 6.5E-05 | 4.0E-04 |  | -0.11 | 8.3E-05 | 6.0E-04 |  | -0.12 | | 4.5E-05 | 5.0E-04 |
| Indole-3-acetate |  | -0.09 | (-0.20 - 0.03) |  | -0.15 | 1.6E-01 | 2.9E-01 |  | -0.15 | 1.5E-01 | 2.7E-01 |  | -0.15 | | 1.9E-01 | 3.7E-01 |
| Isethionate |  | 0.00 | (-0.06 - 0.06) |  | 0 | 9.8E-01 | 9.8E-01 |  | 0 | 9.0E-01 | 9.2E-01 |  | -0.01 | | 6.2E-01 | 7.7E-01 |
| Isocitrate |  | 0.01 | (-0.08 - 0.09) |  | 0.01 | 9.0E-01 | 9.2E-01 |  | 0.01 | 8.3E-01 | 8.7E-01 |  | 0.04 | | 5.1E-01 | 7.0E-01 |
| Kynurenine |  | -0.07 | (-0.11 - -0.02) |  | -0.04 | 7.7E-03 | 2.4E-02 |  | -0.03 | 9.2E-03 | 2.7E-02 |  | -0.01 | | 3.1E-01 | 5.1E-01 |
| Lys |  | -0.07 | (-0.11 - -0.04) |  | -5.13 | 3.5E-05 | 3.0E-04 |  | -5.17 | 3.1E-05 | 3.0E-04 |  | -3.56 | | 8.2E-03 | 3.5E-02 |
| Malate |  | 0.08 | (0.02 - 0.15) |  | 0.18 | 8.5E-03 | 2.6E-02 |  | 0.19 | 5.5E-03 | 1.8E-02 |  | 0.16 | | 3.8E-02 | 1.1E-01 |
| Malonate |  | -0.21 | (-0.36 - -0.05) |  | -0.13 | 7.8E-03 | 2.4E-02 |  | -0.13 | 7.9E-03 | 2.4E-02 |  | -0.11 | | 3.1E-02 | 1.0E-01 |
| Met |  | -0.07 | (-0.14 - 0.01) |  | -0.44 | 8.2E-02 | 1.8E-01 |  | -0.44 | 8.1E-02 | 1.8E-01 |  | -0.44 | | 1.1E-01 | 2.7E-01 |
| Mucate |  | -0.11 | (-0.18 - -0.05) |  | -0.3 | 4.2E-04 | 2.0E-03 |  | -0.3 | 4.0E-04 | 2.0E-03 |  | -0.21 | | 1.7E-02 | 6.0E-02 |
| N-Acetylaspartate |  | 0.04 | (-0.04 - 0.15) |  | 0.01 | 1.6E-01 | 3.0E-01 |  | 0.02 | 1.3E-01 | 2.6E-01 |  | 0.01 | | 2.7E-01 | 4.7E-01 |
| Nicotinamide |  | -0.51 | (-1.01 - 0.00) |  | -0.01 | 2.9E-01 | 4.7E-01 |  | -0.01 | 2.4E-01 | 4.2E-01 |  | 0 | | 4.6E-01 | 6.6E-01 |
| Octanoate |  | 0.13 | (0.05 - 0.20) |  | 0.11 | 1.3E-03 | 5.4E-03 |  | 0.11 | 1.1E-03 | 5.1E-03 |  | 0.1 | | 6.5E-03 | 3.1E-02 |
| Phthalate |  | -0.16 | (-0.32 - -0.08) |  | -0.02 | 1.1E-02 | 3.1E-02 |  | -0.02 | 8.8E-03 | 2.6E-02 |  | -0.01 | | 2.0E-01 | 3.9E-01 |
| Pimelate |  | 0.28 | (0.00 - 0.47) |  | 0.03 | 7.0E-02 | 1.6E-01 |  | 0.03 | 5.5E-02 | 1.3E-01 |  | 0.03 | | 1.1E-01 | 2.7E-01 |
| Pyruvate |  | 0.03 | (-0.04 - 0.09) |  | 0.6 | 4.5E-01 | 6.0E-01 |  | 0.59 | 4.5E-01 | 5.8E-01 |  | 0.95 | | 2.6E-01 | 4.7E-01 |
| Quinate |  | -0.41 | (-0.69 - -0.17) |  | -0.15 | 1.3E-03 | 5.4E-03 |  | -0.16 | 9.4E-04 | 4.4E-03 |  | -0.12 | | 2.5E-02 | 8.4E-02 |
| Symmetrical Dimethylarginine |  | -0.05 | (-0.09 - 0.00) |  | -0.01 | 1.4E-01 | 2.8E-01 |  | -0.01 | 1.4E-01 | 2.6E-01 |  | -0.01 | | 1.2E-01 | 2.7E-01 |
| Terephthalate |  | 0.00 | (-0.11 - 0.07) |  | 0 | 7.1E-01 | 7.7E-01 |  | 0 | 7.0E-01 | 7.8E-01 |  | -0.01 | | 6.8E-01 | 7.9E-01 |
| trans-Aconitate |  | -0.06 | (-0.23 - 0.06) |  | -0.01 | 2.6E-01 | 4.3E-01 |  | -0.01 | 2.7E-01 | 4.3E-01 |  | -0.01 | | 5.0E-01 | 7.0E-01 |
| Triethanolamine |  | 0.02 | (-0.15 - 0.17) |  | 0.01 | 8.9E-01 | 9.2E-01 |  | 0 | 9.1E-01 | 9.2E-01 |  | -0.01 | | 8.0E-01 | 8.3E-01 |
| Trigonelline |  | -0.27 | (-0.45 - -0.09) |  | -0.06 | 1.9E-03 | 7.5E-03 |  | -0.06 | 1.4E-03 | 5.7E-03 |  | -0.07 | | 5.9E-04 | 4.0E-03 |
| Trp |  | 0.01 | (-0.02 - 0.05) |  | 0.18 | 5.3E-01 | 6.7E-01 |  | 0.17 | 5.5E-01 | 6.8E-01 |  | 0.28 | | 3.7E-01 | 5.7E-01 |
| Undecanoate |  | 0.21 | (-0.41 - 0.83) |  | 0.01 | 6.6E-01 | 7.6E-01 |  | 0.01 | 6.7E-01 | 7.7E-01 |  | 0 | | 7.9E-01 | 8.3E-01 |
| Urocanate |  | -0.20 | (-0.52 - 0.09) |  | -0.07 | 1.8E-01 | 3.2E-01 |  | -0.07 | 1.9E-01 | 3.4E-01 |  | -0.04 | | 4.7E-01 | 6.6E-01 |
|  |  | Difference | 95% CI |  | Beta | p | FDR p |  | Beta* | p* | FDR p* |  | Beta*** | | p*** | FDR p*** |
| HDL-cholesterol |  | 0.10 | (0.08 - 0.12) |  | 4.06 | 9.8E-18 |  |  | 4.11 | 3.0E-18 |  |  | 3.75 | | 5.0E-16 |  |
| LDL-cholesterol |  | -0.18 | (-0.27 - -0.09) |  | -3.38 | 1.5E-04 |  |  | -3.45 | 1.0E-04 |  |  | -2.95 | | 1.5E-03 |  |
|  |  |  |  |  |  |  |  |  |  |  |  |  |  | |  |  |
| The associations between metabolites and alcohol intake groups (1: non-drinkers, 2: low 3: middle 4: high alcohol intake groups) in the original population.  Linear regression analysis between each metabolite and alcohol intake group was performed (p-values are shown), then difference between non-drinkers and the high alcohol intake group was calculated using beta of the linear regression analysis.  CI, Confidence interval; FDR, False discovery rate; HDL, High-density lipoprotein; LDL, Low-density lipoprotein * Adjusted for age ** Adjusted for age, BMI, smoking numbers per year, systolic blood pressure, HDL-cholesterol, hemoglobin A1c, daily dietary energy intake and daily physical activity. *** Adjusted for age, BMI, smoking numbers per year, systolic blood pressure, hemoglobin A1c, daily dietary energy intake and daily physical activity. | | | | | | | | | | | | | | | | |
