# Supplementary material for: Metabolomic profiling reveals novel biomarkers of alcohol intake and alcohol-induced liver injury in community-dwelling men
Source: Environ Health Prev Med. 2015 Oct 12;21(1):18–26. doi: 10.1007/s12199-015-0494-y (PMC4693765; doi:10.1007/s12199-015-0494-y)
Supplement: Supplementary file 3 — Supplementary material 3 (DOCX 34 kb) [file 12199_2015_494_MOESM3_ESM.docx]

| **eTable 3.** The associations between metabolites (log-transformed variables) and alcohol intake in the original population. | | | | | | | | | | | | | | | |
| --- | --- | --- | --- | --- | --- | --- | --- | --- | --- | --- | --- | --- | --- | --- | --- |
|  |  |  | | | | | | | | | | | | | |
| **Log-transformed variables** |  | Crude | | | | | |  | Age-adjusted | | |  | Fully-adjusted | | |
|  |  | Fold change | 95% CI |  | Beta | p | FDR p |  | Beta* | p* | FDR p* |  | Beta** | p** | FDR p** |
| 2-Aminobutyrate |  | 1.27 | (1.20-1.35) |  | 0.08 | 4.9E-17 | <.0001 |  | 0.08 | 3.6E-17 | <.0001 |  | 0.07 | 4.1E-13 | <.0001 |
| 2-Hydroxybutyrate |  | 1.16 | (1.09-1.23) |  | 0.05 | 3.9E-05 | 3.0E-04 |  | 0.05 | 3.3E-05 | 3.0E-04 |  | 0.05 | 2.0E-04 | 1.8E-03 |
| 2-Hydroxypentanoate |  | 0.97 | (0.89-1.03) |  | -0.01 | 3.4E-01 | 5.0E-01 |  | -0.01 | 3.3E-01 | 4.8E-01 |  | -0.02 | 1.7E-01 | 3.5E-01 |
| 3-Aminoisobutyrate |  | 0.91 | (0.81-1.06) |  | -0.03 | 2.3E-01 | 3.9E-01 |  | -0.02 | 2.5E-01 | 4.3E-01 |  | -0.03 | 2.7E-01 | 4.7E-01 |
| 3-Hydroxybutyrate |  | 1.06 | (0.94-1.23) |  | 0.02 | 3.5E-01 | 5.0E-01 |  | 0.03 | 2.6E-01 | 4.3E-01 |  | 0.01 | 6.7E-01 | 7.9E-01 |
| 3-Methylhistidine |  | 0.97 | (0.89-1.09) |  | -0.01 | 6.8E-01 | 7.6E-01 |  | -0.01 | 7.4E-01 | 8.0E-01 |  | 0 | 8.0E-01 | 8.3E-01 |
| 4-Methyl-2-oxopentanoate |  | 1.09 | (1.03-1.13) |  | 0.03 | 2.5E-04 | 1.3E-03 |  | 0.03 | 2.9E-04 | 1.6E-03 |  | 0.03 | 1.4E-04 | 1.3E-03 |
| 5-Oxoproline |  | 1 | (0.94-1.03) |  | 0 | 6.7E-01 | 7.6E-01 |  | 0 | 7.5E-01 | 8.0E-01 |  | 0 | 7.4E-01 | 8.2E-01 |
| Ala |  | 0.97 | (0.94-1.00) |  | -0.01 | 1.4E-01 | 2.8E-01 |  | -0.01 | 1.4E-01 | 2.6E-01 |  | 0 | 8.0E-01 | 8.3E-01 |
| Alpha-Aminoadipate |  | 0.91 | (0.86-0.94) |  | -0.03 | 2.3E-04 | 1.3E-03 |  | -0.03 | 2.8E-04 | 1.6E-03 |  | -0.02 | 6.2E-02 | 1.7E-01 |
| Asn |  | 1 | (0.97-1.03) |  | 0 | 6.9E-01 | 7.6E-01 |  | 0 | 6.7E-01 | 7.7E-01 |  | 0 | 7.0E-01 | 7.9E-01 |
| Asp |  | 0.97 | (0.89-1.06) |  | -0.01 | 4.8E-01 | 6.2E-01 |  | -0.01 | 4.5E-01 | 5.8E-01 |  | -0.01 | 6.7E-01 | 7.9E-01 |
| beta-Ala |  | 0.97 | (0.91-1.03) |  | -0.01 | 3.1E-01 | 4.8E-01 |  | -0.01 | 3.0E-01 | 4.6E-01 |  | 0 | 8.2E-01 | 8.5E-01 |
| Betaine |  | 1.03 | (0.97-1.09) |  | 0.01 | 3.5E-01 | 5.0E-01 |  | 0.01 | 3.0E-01 | 4.6E-01 |  | 0 | 8.7E-01 | 8.9E-01 |
| Choline |  | 1.09 | (1.06-1.16) |  | 0.03 | 3.8E-05 | 3.0E-04 |  | 0.03 | 1.8E-05 | 2.0E-04 |  | 0.02 | 9.6E-03 | 3.7E-02 |
| Citrate |  | 0.89 | (0.84-0.97) |  | -0.04 | 2.0E-03 | 7.7E-03 |  | -0.04 | 2.7E-03 | 1.0E-02 |  | -0.03 | 8.8E-03 | 3.5E-02 |
| Citrulline |  | 0.94 | (0.91-1.00) |  | -0.02 | 3.7E-02 | 9.4E-02 |  | -0.02 | 5.2E-02 | 1.3E-01 |  | -0.02 | 3.3E-02 | 1.0E-01 |
| Creatine |  | 1.2 | (1.09-1.27) |  | 0.06 | 1.6E-04 | 1.0E-03 |  | 0.06 | 1.4E-04 | 8.0E-04 |  | 0.05 | 1.2E-03 | 7.6E-03 |
| Creatinine |  | 0.94 | (0.91-1.00) |  | -0.02 | 5.8E-03 | 1.9E-02 |  | -0.02 | 6.9E-03 | 2.2E-02 |  | -0.01 | 7.2E-02 | 1.9E-01 |
| CSSG |  | 0.7 | (0.64-0.76) |  | -0.12 | 6.1E-13 | <.0001 |  | -0.12 | 5.5E-13 | <.0001 |  | -0.12 | 1.6E-12 | <.0001 |
| Cystine |  | 0.97 | (0.94-1.00) |  | -0.01 | 8.3E-02 | 1.8E-01 |  | -0.01 | 1.1E-01 | 2.3E-01 |  | 0 | 4.3E-01 | 6.4E-01 |
| Glu |  | 1.09 | (1.00-1.16) |  | 0.03 | 2.6E-02 | 7.2E-02 |  | 0.03 | 2.9E-02 | 7.9E-02 |  | 0.05 | 1.4E-05 | 2.0E-04 |
| Gly |  | 0.97 | (0.94-1.03) |  | -0.01 | 3.8E-01 | 5.3E-01 |  | -0.01 | 3.6E-01 | 5.1E-01 |  | -0.01 | 2.9E-01 | 4.9E-01 |
| Glycerophosphorylcholine |  | 1.16 | (1.09-1.23) |  | 0.05 | 2.6E-06 | <.0001 |  | 0.05 | 3.1E-06 | <.0001 |  | 0.04 | 1.8E-03 | 9.9E-03 |
| His |  | 1.03 | (1.00-1.06) |  | 0.01 | 6.2E-02 | 1.5E-01 |  | 0.01 | 7.1E-02 | 1.6E-01 |  | 0.01 | 7.4E-03 | 3.4E-02 |
| Hydroxyproline |  | 1.09 | (1.03-1.16) |  | 0.03 | 1.2E-03 | 5.4E-03 |  | 0.03 | 1.2E-03 | 5.1E-03 |  | 0.02 | 2.7E-02 | 8.8E-02 |
| Ile |  | 0.89 | (0.86-0.94) |  | -0.04 | 1.0E-08 | <.0001 |  | -0.04 | 1.3E-08 | <.0001 |  | -0.02 | 2.6E-04 | 2.2E-03 |
| Lactate |  | 0.97 | (0.91-1.03) |  | -0.01 | 3.5E-01 | 5.0E-01 |  | -0.01 | 3.6E-01 | 5.1E-01 |  | -0.01 | 4.1E-01 | 6.3E-01 |
| Leu |  | 0.94 | (0.91-0.97) |  | -0.02 | 3.5E-04 | 1.8E-03 |  | -0.02 | 3.5E-04 | 1.8E-03 |  | -0.01 | 7.7E-02 | 2.0E-01 |
| N,N-Dimethylglycine |  | 0.91 | (0.86-0.97) |  | -0.03 | 4.0E-03 | 1.4E-02 |  | -0.03 | 4.3E-03 | 1.5E-02 |  | -0.03 | 6.5E-03 | 3.1E-02 |
| o-Acetylcarnitine |  | 0.91 | (0.76-1.09) |  | -0.03 | 3.2E-01 | 4.9E-01 |  | -0.03 | 3.1E-01 | 4.6E-01 |  | -0.02 | 5.6E-01 | 7.3E-01 |
| Ornithine |  | 0.86 | (0.84-0.89) |  | -0.05 | 3.4E-13 | <.0001 |  | -0.05 | 4.9E-13 | <.0001 |  | -0.04 | 6.8E-08 | <.0001 |
| Pelargonate |  | 0.97 | (0.89-1.06) |  | -0.01 | 5.5E-01 | 6.9E-01 |  | -0.01 | 5.4E-01 | 6.8E-01 |  | -0.01 | 4.8E-01 | 6.8E-01 |
| Phe |  | 1 | (0.97-1.03) |  | 0 | 7.1E-01 | 7.7E-01 |  | 0 | 5.8E-01 | 7.0E-01 |  | 0.01 | 2.8E-01 | 4.8E-01 |
| Pipecolate |  | 1.72 | (1.57-1.88) |  | 0.18 | 2.9E-28 | <.0001 |  | 0.18 | 2.6E-29 | <.0001 |  | 0.16 | 7.4E-21 | <.0001 |
| Pro |  | 0.97 | (0.91-1.00) |  | -0.01 | 9.4E-02 | 2.0E-01 |  | -0.02 | 9.0E-02 | 2.0E-01 |  | -0.01 | 5.6E-01 | 7.3E-01 |
| Proline betaine |  | 0.86 | (0.72-1.00) |  | -0.05 | 4.0E-02 | 1.0E-01 |  | -0.05 | 5.2E-02 | 1.3E-01 |  | -0.04 | 2.0E-01 | 3.8E-01 |
| Sarcosine |  | 1.06 | (1.00-1.16) |  | 0.02 | 9.7E-02 | 2.0E-01 |  | 0.02 | 9.6E-02 | 2.0E-01 |  | 0.02 | 1.9E-01 | 3.7E-01 |
| Ser |  | 0.94 | (0.91-1.00) |  | -0.02 | 2.5E-02 | 7.2E-02 |  | -0.02 | 2.7E-02 | 7.7E-02 |  | -0.01 | 1.2E-01 | 2.7E-01 |
| Succinate |  | 1 | (0.94-1.03) |  | 0 | 6.8E-01 | 7.6E-01 |  | 0 | 7.5E-01 | 8.0E-01 |  | 0 | 6.8E-01 | 7.9E-01 |
| Taurine |  | 0.94 | (0.91-0.97) |  | -0.02 | 2.3E-03 | 8.5E-03 |  | -0.02 | 2.5E-03 | 9.8E-03 |  | -0.02 | 3.3E-03 | 1.7E-02 |
| Thr |  | 1.06 | (1.03-1.09) |  | 0.02 | 1.2E-03 | 5.4E-03 |  | 0.02 | 1.2E-03 | 5.1E-03 |  | 0.02 | 1.3E-02 | 4.7E-02 |
| Threonate |  | 0.91 | (0.86-0.97) |  | -0.03 | 3.2E-03 | 1.1E-02 |  | -0.03 | 3.4E-03 | 1.2E-02 |  | -0.03 | 1.2E-02 | 4.4E-02 |
| Trimethylamine N-oxide |  | 1.13 | (0.94-1.35) |  | 0.04 | 1.5E-01 | 2.9E-01 |  | 0.04 | 1.2E-01 | 2.4E-01 |  | 0.06 | 5.5E-02 | 1.6E-01 |
| Tyr |  | 1.06 | (1.03-1.09) |  | 0.02 | 2.3E-04 | 1.3E-03 |  | 0.02 | 1.0E-04 | 7.0E-04 |  | 0.02 | 6.3E-05 | 7.0E-04 |
| Urate |  | 0.94 | (0.81-1.13) |  | -0.02 | 5.9E-01 | 7.2E-01 |  | -0.02 | 5.7E-01 | 7.0E-01 |  | 0 | 9.3E-01 | 9.4E-01 |
| Urea |  | 0.97 | (0.89-1.03) |  | -0.01 | 2.2E-01 | 3.8E-01 |  | -0.01 | 2.7E-01 | 4.3E-01 |  | -0.01 | 6.7E-01 | 7.9E-01 |
| Uridine |  | 0.97 | (0.94-1.03) |  | -0.01 | 2.9E-01 | 4.7E-01 |  | -0.01 | 2.9E-01 | 4.5E-01 |  | -0.01 | 3.8E-01 | 5.9E-01 |
| Val |  | 0.91 | (0.89-0.94) |  | -0.03 | 2.5E-07 | <.0001 |  | -0.03 | 3.0E-07 | <.0001 |  | -0.02 | 1.7E-03 | 9.8E-03 |
|  |  | Fold change | 95% CI |  | Beta | p | FDR p |  | Beta* | p* | FDR p* |  | Beta** | p*** | FDR p*** |
| Triglyceride |  | 1.03 | (0.94-1.13) |  | 0.01 | 3.4E-01 |  |  | 0.01 | 4.1E-01 |  |  | 0.01 | 5.8E-01 |  |
|  |  |  |  |  |  |  |  |  |  |  |  |  |  |  |  |
| The associations between metabolites and alcohol intake groups (1: non-drinkers, 2: low 3: middle 4: high alcohol intake groups) in the original population.  Linear regression analysis between each metabolite and alcohol intake group was performed (p-values are shown), then fold change between non-drinkers and the high alcohol intake group was calculated using beta of the linear regression analysis.  CI, Confidence interval; CSSG, Cysteine-glutathione disulfide; FDR, False discovery rate; HDL, High-density lipoprotein * Adjusted for age ** Adjusted for age, BMI, smoking numbers per year, systolic blood pressure, HDL-cholesterol, hemoglobin A1c, daily dietary energy intake and daily physical activity.  *** Adjusted for age, BMI, smoking numbers per year, systolic blood pressure, hemoglobin A1c, daily dietary energy intake and daily physical activity. | | | | | | | | | | | | | | | |
