# Supplementary material for: Metabolomic profiling reveals novel biomarkers of alcohol intake and alcohol-induced liver injury in community-dwelling men
Source: Environ Health Prev Med. 2015 Oct 12;21(1):18–26. doi: 10.1007/s12199-015-0494-y (PMC4693765; doi:10.1007/s12199-015-0494-y)
Supplement: Supplementary file 4 — Supplementary material 4 (DOCX 31 kb) [file 12199_2015_494_MOESM4_ESM.docx]

| **eTable 4.** The association between alcohol-related plasma metabolites and serum γ-GTP | | | | | | | | | | |  |
| --- | --- | --- | --- | --- | --- | --- | --- | --- | --- | --- | --- |
|  |  |  |  |  |  |  |  |  |  |  | |
|  |  |  |  |  |  |  |  |  |  |  | |
| **High alcohol intake** |  | **original population** | | | | | | | | | |
|  |  | Age-adjusted* | | | | |  | Fully-adjusted** | | | |
|  |  | Fold change | 95% CI | B | p | FDR p |  | B | p | FDR p | |
| CSSG (log) |  | 0.66 | (0.60 - 0.73) | -0.41 | 2.4E-14 | <.0001 |  | -0.41 | 1.2E-14 | <.0001 | |
| Guanidinosuccinate |  | 0.68 | (0.62 - 0.76) | -0.38 | 4.6E-12 | <.0001 |  | -0.35 | 2.7E-10 | <.0001 | |
| Gln |  | 0.70 | (0.63 - 0.77) | -0.36 | 7.2E-11 | <.0001 |  | -0.36 | 6.2E-11 | <.0001 | |
| Thr (log) |  | 1.32 | (1.18 - 1.47) | 0.28 | 7.3E-07 | <.0001 |  | 0.25 | 5.8E-06 | <.0001 | |
| 2-Aminobutyrate (log) |  | 0.82 | (0.73 - 0.91) | -0.20 | 4.4E-04 | 1.8E-03 |  | -0.19 | 4.8E-04 | 1.9E-03 | |
| Carnitine |  | 1.20 | (1.08 - 1.34) | 0.18 | 1.2E-03 | 4.0E-03 |  | 0.18 | 1.2E-03 | 4.1E-03 | |
| Hippurate |  | 0.85 | (0.76 - 0.95) | -0.16 | 4.3E-03 | 1.2E-02 |  | -0.14 | 1.1E-02 | 3.3E-02 | |
| Choline (log) |  | 1.16 | (1.03 - 1.29) | 0.14 | 1.3E-02 | 3.3E-02 |  | 0.12 | 3.2E-02 | 8.0E-02 | |
| 2-Hydroxybutyrate (log) |  | 1.14 | (1.02 - 1.27) | 0.13 | 2.4E-02 | 5.3E-02 |  | 0.12 | 5.1E-02 | 1.0E-01 | |
| Pipecolate (log) |  | 0.89 | (0.80 - 1.00) | -0.12 | 4.5E-02 | 9.0E-02 |  | -0.11 | 5.2E-02 | 1.0E-01 | |
| Arg |  | 0.92 | (0.82 - 1.03) | -0.09 | 1.3E-01 | 2.3E-01 |  | -0.07 | 2.1E-01 | 3.2E-01 | |
| Threonate (log) |  | 0.93 | (0.83 - 1.04) | -0.07 | 2.0E-01 | 3.3E-01 |  | -0.04 | 4.6E-01 | 5.2E-01 | |
| Creatine (log) |  | 1.07 | (0.96 - 1.20) | 0.07 | 2.4E-01 | 3.6E-01 |  | 0.06 | 2.5E-01 | 3.3E-01 | |
| Trigonelline |  | 0.94 | (0.84 - 1.06) | -0.06 | 3.2E-01 | 4.6E-01 |  | -0.04 | 4.7E-01 | 5.2E-01 | |
| Leu |  | 0.95 | (0.85 - 1.07) | -0.05 | 4.0E-01 | 4.9E-01 |  | -0.09 | 1.1E-01 | 1.9E-01 | |
| Val (log) |  | 0.95 | (0.85 - 1.07) | -0.05 | 4.1E-01 | 4.9E-01 |  | -0.09 | 1.2E-01 | 1.9E-01 | |
| Creatinine |  | 0.96 | (0.85 - 1.07) | -0.05 | 4.3E-01 | 4.9E-01 |  | -0.06 | 2.9E-01 | 3.7E-01 | |
| Glycerophosphorylcholine (log) |  | 1.04 | (0.93 - 1.17) | 0.04 | 4.6E-01 | 4.9E-01 |  | 0.07 | 2.4E-01 | 3.3E-01 | |
| Ornithine (log) |  | 1.04 | (0.93 - 1.17) | 0.04 | 4.6E-01 | 4.9E-01 |  | 0.04 | 5.3E-01 | 5.6E-01 | |
| Ile (log) |  | 1.02 | (0.91 - 1.14) | 0.02 | 7.2E-01 | 7.2E-01 |  | -0.03 | 6.0E-01 | 6.0E-01 | |
| Glu/Gln ratio |  | 1.82 | (1.67 - 1.97) | 0.60 | 1.0E-30 |  |  | 0.59 | 1.0E-30 |  | |
|  |  |  |  |  |  |  |  |  |  |  | |
|  |  | **replication population** | | | | | | | | | |
|  |  | Age-adjusted* | | | | |  | Fully-adjusted** | | | |
|  |  | Fold change | 95% CI | B | p |  |  | B | p |  | |
| CSSG (log) |  | 0.55 | (0.46 - 0.67) | -0.59 | 1.3E-07 |  |  | -0.62 | 3.2E-07 |  | |
| Guanidinosuccinate |  | 0.52 | (0.44 - 0.63) | -0.65 | 5.1E-09 |  |  | -0.70 | 1.3E-08 |  | |
| Gln |  | 0.81 | (0.63 - 1.04) | -0.21 | 9.3E-02 |  |  | -0.19 | 1.5E-01 |  | |
| Thr (log) |  | 1.39 | (1.09 - 1.77) | 0.33 | 8.5E-03 |  |  | 0.47 | 9.9E-04 |  | |
| 2-Aminobutyrate (log) |  | 1.04 | (0.80 - 1.35) | 0.04 | 7.7E-01 |  |  | 0.04 | 8.0E-01 |  | |
| Carnitine |  | 1.59 | (1.27 - 1.98) | 0.46 | 1.1E-04 |  |  | 0.45 | 4.2E-04 |  | |
| Hippurate |  | 0.91 | (0.69 - 1.19) | -0.10 | 4.8E-01 |  |  | -0.17 | 2.4E-01 |  | |
| Choline (log) |  | 1.38 | (1.09 - 1.76) | 0.33 | 9.1E-03 |  |  | 0.32 | 1.2E-02 |  | |
| Glu/Gln ratio |  | 1.17 | (1.04 - 1.31) | 0.15 | 9.8E-03 |  |  | 0.15 | 1.5E-02 |  | |
|  |  |  |  |  |  |  |  |  |  |  | |
|  |  |  |  |  |  |  |  |  |  |  | |
| **Non-drinkers** |  | **original population** | | | | | | | | | |
|  |  | Age-adjusted* | | | | |  | Fully-adjusted** | | | |
|  |  | Fold change | 95% CI | B | p | FDR p |  | B | p | FDR p | |
| CSSG (log) |  | 0.79 | (0.75 - 0.84) | -0.23 | 2.0E-13 | <.0001 |  | -0.21 | 3.2E-11 | <.0001 | |
| Guanidinosuccinate |  | 0.96 | (0.90 - 1.02) | -0.04 | 2.0E-01 | 3.1E-01 |  | -0.04 | 1.7E-01 | 3.4E-01 | |
| Gln |  | 0.96 | (0.90 - 1.03) | -0.04 | 2.6E-01 | 3.4E-01 |  | -0.04 | 2.5E-01 | 3.8E-01 | |
| Thr (log) |  | 0.93 | (0.87 - 0.99) | -0.07 | 2.7E-02 | 6.7E-02 |  | -0.07 | 3.3E-02 | 9.5E-02 | |
| 2-Aminobutyrate (log) |  | 1.01 | (0.94 - 1.08) | 0.01 | 7.9E-01 | 8.6E-01 |  | 0.00 | 9.5E-01 | 9.5E-01 | |
| Carnitine |  | 1.10 | (1.03 - 1.17) | 0.09 | 6.3E-03 | 2.1E-02 |  | 0.08 | 8.6E-03 | 3.4E-02 | |
| Hippurate |  | 0.96 | (0.90 - 1.02) | -0.04 | 1.9E-01 | 3.1E-01 |  | -0.04 | 2.3E-01 | 3.8E-01 | |
| Choline (log) |  | 1.04 | (0.98 - 1.12) | 0.04 | 2.0E-01 | 3.1E-01 |  | 0.04 | 2.3E-01 | 3.8E-01 | |
| 2-Hydroxybutyrate (log) |  | 1.15 | (1.08 - 1.23) | 0.14 | 1.6E-05 | 2.0E-04 |  | 0.12 | 4.0E-04 | 4.0E-03 | |
| Pipecolate (log) |  | 0.96 | (0.90 - 1.03) | -0.04 | 2.2E-01 | 3.1E-01 |  | -0.05 | 1.5E-01 | 3.4E-01 | |
| Arg |  | 0.92 | (0.86 - 0.98) | -0.08 | 1.4E-02 | 3.9E-02 |  | -0.07 | 3.3E-02 | 9.5E-02 | |
| Threonate (log) |  | 0.94 | (0.88 - 1.01) | -0.06 | 8.7E-02 | 1.7E-01 |  | -0.03 | 3.2E-01 | 4.3E-01 | |
| Creatine (log) |  | 1.07 | (1.00 - 1.14) | 0.06 | 5.8E-02 | 1.3E-01 |  | 0.03 | 3.1E-01 | 4.3E-01 | |
| Trigonelline |  | 1.00 | (0.93 - 1.07) | 0.00 | 9.4E-01 | 9.4E-01 |  | 0.03 | 4.3E-01 | 5.4E-01 | |
| Leu |  | 1.13 | (1.06 - 1.20) | 0.12 | 2.6E-04 | 1.3E-03 |  | 0.09 | 8.2E-03 | 3.4E-02 | |
| Val (log) |  | 1.11 | (1.04 - 1.18) | 0.10 | 2.5E-03 | 1.0E-02 |  | 0.06 | 5.7E-02 | 1.4E-01 | |
| Creatinine |  | 1.03 | (0.96 - 1.10) | 0.03 | 4.2E-01 | 4.9E-01 |  | 0.02 | 4.8E-01 | 5.6E-01 | |
| Glycerophosphorylcholine (log) |  | 0.99 | (0.93 - 1.06) | -0.01 | 8.1E-01 | 8.6E-01 |  | 0.00 | 9.4E-01 | 9.5E-01 | |
| Ornithine (log) |  | 0.97 | (0.91 - 1.04) | -0.03 | 4.1E-01 | 4.9E-01 |  | -0.02 | 5.6E-01 | 6.2E-01 | |
| Ile (log) |  | 1.13 | (1.06 - 1.20) | 0.12 | 2.4E-04 | 1.3E-03 |  | 0.09 | 6.7E-03 | 3.4E-02 | |
| Glu/Gln ratio |  | 1.24 | (1.17 - 1.31) | 0.21 | 2.2E-11 |  |  | 0.19 | 2.5E-07 |  | |
|  |  |  |  |  |  |  |  |  |  |  | |
|  |  | **replication population** | | | | | | | | | |
|  |  | Age-adjusted* | | | | |  | Fully-adjusted** | | | |
|  |  | Fold change | 95% CI | B | p |  |  | B | p |  | |
| CSSG (log) |  | 0.72 | (0.64 - 0.81) | -0.33 | 9.1E-07 |  |  | -0.31 | 2.8E-05 |  | |
| Guanidinosuccinate |  | 0.91 | (0.77 - 1.07) | -0.09 | 2.6E-01 |  |  | -0.06 | 5.4E-01 |  | |
| Gln |  | 1.04 | (0.90 - 1.21) | 0.04 | 5.7E-01 |  |  | 0.04 | 6.2E-01 |  | |
| Thr (log) |  | 0.96 | (0.83 - 1.12) | -0.04 | 6.1E-01 |  |  | -0.10 | 1.9E-01 |  | |
| 2-Aminobutyrate (log) |  | 1.06 | (0.91 - 1.23) | 0.06 | 4.3E-01 |  |  | 0.01 | 9.1E-01 |  | |
| Carnitine |  | 1.02 | (0.88 - 1.19) | 0.02 | 8.1E-01 |  |  | 0.00 | 9.5E-01 |  | |
| Hippurate |  | 0.93 | (0.80 - 1.08) | -0.08 | 3.1E-01 |  |  | -0.06 | 4.3E-01 |  | |
| Choline (log) |  | 0.98 | (0.84 - 1.14) | -0.02 | 8.2E-01 |  |  | -0.09 | 2.5E-01 |  | |
| Glu/Gln ratio |  | 1.23 | (1.07 - 1.41) | 0.21 | 3.7E-03 |  |  | 0.18 | 2.6E-02 |  | |
|  |  |  |  |  |  |  |  |  |  |  | |
| The associations between alcohol-related plasma metabolites and serum γ-GTP in the high alcohol intake group and non-drinkers were shown. Linear regression analysis between each alcohol-related metabolite (log-transformed if necessary) and γ-GTP (log-transformed) was performed (Standardized betas and p-values are shown), then we calculated fold change and 95% confidence interval of serum γ-GTP, per one standard deviation increase in each metabolite using standardized beta of the linear regression analysis. | | | | | | | | | | |  |
| Replication analyses were performed for metabolites with less than 0.05 false discovery rate p-values in the original population and glutamine/glutamine ratio. | | | | | | | | | | |  |
| CI, Confidence interval ; CSSG, Cysteine-glutathione disulfide; γ-GTP, gamma-glutamyl transpeptidase | | | | | | | | | | |  |
| * Adjusted for age | | | | | | | | | | |  |
| ** Adjusted for age, BMI, smoking numbers per year, systolic blood pressure, HDL-cholesterol, hemoglobin A1c, daily dietary energy intake and daily physical activity. | | | | | | | | | | |  |
