# Supplementary material for: Metabolomic profiling reveals novel biomarkers of alcohol intake and alcohol-induced liver injury in community-dwelling men
Source: Environ Health Prev Med. 2015 Oct 12;21(1):18–26. doi: 10.1007/s12199-015-0494-y (PMC4693765; doi:10.1007/s12199-015-0494-y)
Supplement: Supplementary file 6 — Supplementary material 6 (DOCX 30 kb) [file 12199_2015_494_MOESM6_ESM.docx]

| **eTable 6.** The association between alcohol-related plasma metabolites and serum ALT | | | | | | | | | | |
| --- | --- | --- | --- | --- | --- | --- | --- | --- | --- | --- |
|  |  |  |  |  |  |  |  |  |  |  |
|  |  |  |  |  |  |  |  |  |  |  |
| **High alcohol intake** |  | **original population** | | | | | | | | |
|  |  | Age-adjusted* | | | | |  | Fully-adjusted** | | |
|  |  | Fold change | 95% CI | B | p | FDR p |  | B | p | FDR p |
| CSSG (log) |  | 0.90 | (0.85 - 0.95) | -0.11 | 1.8E-04 | 2.3E-03 |  | -0.10 | 3.5E-04 | 3.5E-03 |
| Guanidinosuccinate |  | 0.90 | (0.85 - 0.95) | -0.11 | 2.3E-04 | 2.3E-03 |  | -0.09 | 2.3E-03 | 1.6E-02 |
| Gln |  | 0.90 | (0.85 - 0.96) | -0.10 | 5.2E-04 | 3.5E-03 |  | -0.10 | 2.3E-04 | 3.5E-03 |
| Pipecolate (log) |  | 0.92 | (0.86 - 0.97) | -0.09 | 3.0E-03 | 1.5E-02 |  | -0.07 | 1.0E-02 | 4.0E-02 |
| 2-Hydroxybutyrate (log) |  | 1.09 | (1.03 - 1.15) | 0.08 | 4.5E-03 | 1.8E-02 |  | 0.06 | 5.0E-02 | 1.3E-01 |
| Creatine (log) |  | 1.08 | (1.02 - 1.15) | 0.08 | 6.4E-03 | 1.9E-02 |  | 0.08 | 4.5E-03 | 2.2E-02 |
| Thr (log) |  | 1.08 | (1.02 - 1.15) | 0.08 | 6.6E-03 | 1.9E-02 |  | 0.06 | 2.1E-02 | 6.6E-02 |
| Carnitine |  | 1.06 | (1 - 1.12) | 0.06 | 4.8E-02 | 1.2E-01 |  | 0.06 | 2.3E-02 | 6.6E-02 |
| Arg |  | 0.95 | (0.89 - 1.01) | -0.05 | 7.5E-02 | 1.7E-01 |  | -0.03 | 2.6E-01 | 5.2E-01 |
| Trigonelline |  | 0.95 | (0.9 - 1.01) | -0.05 | 1.1E-01 | 2.1E-01 |  | -0.03 | 3.4E-01 | 5.5E-01 |
| Ile (log) |  | 1.05 | (0.99 - 1.11) | 0.05 | 1.2E-01 | 2.1E-01 |  | 0.00 | 9.7E-01 | 9.7E-01 |
| 2-Aminobutyrate (log) |  | 0.96 | (0.9 - 1.01) | -0.04 | 1.4E-01 | 2.4E-01 |  | -0.04 | 1.1E-01 | 2.5E-01 |
| Ornithine (log) |  | 1.04 | (0.98 - 1.1) | 0.04 | 2.3E-01 | 3.5E-01 |  | 0.03 | 3.6E-01 | 5.5E-01 |
| Leu |  | 1.04 | (0.98 - 1.1) | 0.03 | 2.4E-01 | 3.5E-01 |  | -0.01 | 8.6E-01 | 9.6E-01 |
| Hippurate |  | 0.97 | (0.91 - 1.03) | -0.03 | 2.9E-01 | 3.8E-01 |  | -0.02 | 5.1E-01 | 6.5E-01 |
| Val (log) |  | 1.02 | (0.96 - 1.08) | 0.02 | 4.9E-01 | 6.2E-01 |  | -0.03 | 3.4E-01 | 5.5E-01 |
| Creatinine |  | 1.02 | (0.96 - 1.08) | 0.02 | 5.9E-01 | 7.0E-01 |  | 0.00 | 9.1E-01 | 9.6E-01 |
| Choline (log) |  | 0.99 | (0.93 - 1.05) | -0.01 | 7.8E-01 | 8.7E-01 |  | -0.01 | 7.2E-01 | 8.5E-01 |
| Threonate (log) |  | 1.00 | (0.94 - 1.06) | 0.00 | 9.4E-01 | 9.7E-01 |  | 0.02 | 5.2E-01 | 6.5E-01 |
| Glycerophosphorylcholine (log) |  | 1.00 | (0.94 - 1.06) | 0.00 | 9.7E-01 | 9.7E-01 |  | 0.02 | 3.9E-01 | 5.5E-01 |
| Glu/Gln ratio |  | 1.25 | (1.19 - 1.32) | 0.22 | 2.3E-15 |  |  | 0.20 | 7.9E-13 |  |
|  |  |  |  |  |  |  |  |  |  |  |
|  |  | **replication population** | | | | | | | | |
|  |  | Age-adjusted* | | | | |  | Fully-adjusted** | | |
|  |  | Fold change | 95% CI | B | p |  |  | B | p |  |
| CSSG (log) |  | 0.84 | (0.72 - 0.98) | -0.17 | 2.7E-02 |  |  | -0.18 | 3.9E-02 |  |
| Guanidinosuccinate |  | 0.79 | (0.68 - 0.91) | -0.24 | 2.3E-03 |  |  | -0.25 | 3.4E-03 |  |
| Gln |  | 0.89 | (0.77 - 1.05) | -0.11 | 1.6E-01 |  |  | -0.10 | 2.2E-01 |  |
| Pipecolate (log) |  | 1.02 | (0.86 - 1.2) | 0.02 | 8.6E-01 |  |  | 0.01 | 9.1E-01 |  |
| 2-Hydroxybutyrate (log) |  | 1.23 | (1.06 - 1.42) | 0.21 | 7.2E-03 |  |  | 0.19 | 2.4E-02 |  |
| Creatine (log) |  | 1.12 | (0.95 - 1.31) | 0.11 | 1.7E-01 |  |  | 0.13 | 1.3E-01 |  |
| Thr (log) |  | 1.06 | (0.9 - 1.24) | 0.05 | 5.0E-01 |  |  | 0.12 | 2.1E-01 |  |
| Glu/Gln ratio |  | 1.25 | (1.08 - 1.45) | 0.23 | 3.0E-03 |  |  | 0.22 | 7.8E-03 |  |
|  |  |  |  |  |  |  |  |  |  |  |
|  |  |  |  |  |  |  |  |  |  |  |
| **Non-drinkers** |  | **original population** | | | | | | | | |
|  |  | Age-adjusted* | | | | |  | Fully-adjusted** | | |
|  |  | Fold change | 95% CI | B | p | FDR p |  | B | p | FDR p |
| CSSG (log) |  | 0.91 | (0.86 - 0.96) | -0.09 | 7.1E-04 | 3.5E-03 |  | -0.06 | 1.8E-02 | 7.3E-02 |
| Guanidinosuccinate |  | 0.99 | (0.93 - 1.05) | -0.01 | 7.0E-01 | 7.1E-01 |  | -0.01 | 7.0E-01 | 7.4E-01 |
| Gln |  | 0.95 | (0.9 - 1) | -0.05 | 6.6E-02 | 1.3E-01 |  | -0.05 | 6.4E-02 | 2.1E-01 |
| Pipecolate (log) |  | 0.98 | (0.92 - 1.03) | -0.02 | 4.3E-01 | 5.0E-01 |  | -0.02 | 4.9E-01 | 7.0E-01 |
| 2-Hydroxybutyrate (log) |  | 1.13 | (1.08 - 1.2) | 0.13 | 5.2E-06 | 1.0E-04 |  | 0.09 | 6.7E-04 | 4.5E-03 |
| Creatine (log) |  | 1.13 | (1.07 - 1.19) | 0.12 | 1.6E-05 | 2.0E-04 |  | 0.10 | 3.0E-04 | 3.0E-03 |
| Thr (log) |  | 0.93 | (0.88 - 0.98) | -0.08 | 6.7E-03 | 2.7E-02 |  | -0.07 | 5.8E-03 | 2.9E-02 |
| Carnitine |  | 1.03 | (0.97 - 1.09) | 0.03 | 3.4E-01 | 4.2E-01 |  | 0.02 | 4.3E-01 | 6.6E-01 |
| Arg |  | 0.95 | (0.9 - 1) | -0.05 | 7.3E-02 | 1.3E-01 |  | -0.03 | 2.0E-01 | 4.0E-01 |
| Trigonelline |  | 0.93 | (0.88 - 0.98) | -0.07 | 1.1E-02 | 3.7E-02 |  | -0.04 | 1.2E-01 | 3.5E-01 |
| Ile (log) |  | 1.04 | (0.98 - 1.1) | 0.04 | 2.1E-01 | 2.8E-01 |  | -0.01 | 7.9E-01 | 7.9E-01 |
| 2-Aminobutyrate (log) |  | 1.06 | (1 - 1.12) | 0.06 | 4.2E-02 | 1.0E-01 |  | 0.04 | 1.6E-01 | 4.0E-01 |
| Ornithine (log) |  | 0.89 | (0.84 - 0.94) | -0.12 | 3.0E-05 | 2.0E-04 |  | -0.11 | 6.5E-05 | 1.3E-03 |
| Leu |  | 1.06 | (1 - 1.12) | 0.06 | 4.6E-02 | 1.0E-01 |  | 0.02 | 5.8E-01 | 7.0E-01 |
| Hippurate |  | 0.96 | (0.9 - 1.01) | -0.04 | 1.2E-01 | 1.8E-01 |  | -0.04 | 1.9E-01 | 4.0E-01 |
| Val (log) |  | 1.06 | (1 - 1.12) | 0.06 | 4.5E-02 | 1.0E-01 |  | 0.01 | 6.1E-01 | 7.0E-01 |
| Creatinine |  | 0.98 | (0.93 - 1.04) | -0.02 | 4.5E-01 | 5.0E-01 |  | -0.03 | 2.4E-01 | 4.1E-01 |
| Choline (log) |  | 0.99 | (0.93 - 1.05) | -0.01 | 7.1E-01 | 7.1E-01 |  | -0.01 | 6.3E-01 | 7.0E-01 |
| Threonate (log) |  | 0.96 | (0.91 - 1.02) | -0.04 | 1.6E-01 | 2.3E-01 |  | -0.02 | 5.7E-01 | 7.0E-01 |
| Glycerophosphorylcholine (log) |  | 0.96 | (0.91 - 1.01) | -0.04 | 1.2E-01 | 1.8E-01 |  | -0.03 | 2.5E-01 | 4.1E-01 |
| Glu/Gln ratio |  | 1.16 | (1.1 - 1.22) | 0.15 | 4.7E-08 |  |  | 0.09 | 2.0E-03 |  |
|  |  |  |  |  |  |  |  |  |  |  |
|  |  | **replication population** | | | | | | | | |
|  |  | Age-adjusted* | | | | |  | Fully-adjusted** | | |
|  |  | Fold change | 95% CI | B | p |  |  | B | p |  |
| CSSG (log) |  | 0.76 | (0.65 - 0.89) | -0.28 | 9.2E-04 |  |  | -0.28 | 8.4E-04 |  |
| Guanidinosuccinate |  | 0.93 | (0.77 - 1.12) | -0.07 | 4.4E-01 |  |  | -0.02 | 8.3E-01 |  |
| Gln |  | 0.95 | (0.8 - 1.13) | -0.05 | 5.9E-01 |  |  | -0.04 | 6.6E-01 |  |
| Pipecolate (log) |  | 0.95 | (0.8 - 1.13) | -0.06 | 5.3E-01 |  |  | -0.05 | 5.1E-01 |  |
| 2-Hydroxybutyrate (log) |  | 1.13 | (0.95 - 1.33) | 0.12 | 1.6E-01 |  |  | 0.07 | 3.6E-01 |  |
| Creatine (log) |  | 1.07 | (0.9 - 1.27) | 0.07 | 4.4E-01 |  |  | 0.04 | 6.4E-01 |  |
| Thr (log) |  | 0.89 | (0.75 - 1.06) | -0.11 | 1.9E-01 |  |  | -0.19 | 1.6E-02 |  |
| Glu/Gln ratio |  | 1.25 | (1.07 - 1.47) | 0.22 | 7.0E-03 |  |  | 0.20 | 2.4E-02 |  |
|  |  |  |  |  |  |  |  |  |  |  |
| The associations between alcohol-related plasma metabolites and serum ALT in the high alcohol intake group and non-drinkers were shown. Linear regression analysis between each alcohol-related metabolite (log-transformed if necessary) and ALT (log-transformed) was performed (Standardized betas and p-values are shown), then we calculated fold change and 95% confidence interval of serum ALT, per one standard deviation increase in each metabolite using standardized beta of the linear regression analysis. | | | | | | | | | | |
| Replication analyses were performed for metabolites with less than 0.05 false discovery rate p-values in the original population and glutamine/glutamine ratio. | | | | | | | | | | |
| CI, Confidence interval; CSSG, Cysteine-glutathione disulfide; ALT, alanine aminotransferase | | | | | | | | | | |
| * Adjusted for age | | | | | | | | | | |
| ** Adjusted for age, BMI, smoking numbers per year, systolic blood pressure, HDL-cholesterol, hemoglobin A1c, daily dietary energy intake and daily physical activity. | | | | | | | | | | |
